# Expression graph network framework for biomarker discovery

**DOI:** 10.1093/bib/bbaf559

**Published:** 2025-10-27

**Authors:** Yang Liu, Jason Huse, Kasthuri Kannan

**Affiliations:** Department of Translational Molecular Pathology, University of Texas MD Anderson Cancer Center, 2130 W Holcombe Blvd, Texas 77030, United States; Department of Biostatistics and Data Science, University of Texas Health Science Center at Houston, 1200 Pressler Street, Texas 77030, United States; Department of Translational Molecular Pathology, University of Texas MD Anderson Cancer Center, 2130 W Holcombe Blvd, Texas 77030, United States; Department of Pathology, University of Texas MD Anderson Cancer Center, 1515 Holcombe Blvd, Texas 77030, United States; Department of Translational Molecular Pathology, University of Texas MD Anderson Cancer Center, 2130 W Holcombe Blvd, Texas 77030, United States

**Keywords:** biomarker, graph neural network, EGNF

## Abstract

Biomarker discovery for complex diseases, such as cancer, hinges on uncovering molecular signatures that capture intricate, interconnected relationships within biological data—a challenge that traditional statistical and machine learning methods often fail to meet due to the complexity of high-dimensional gene expression profiles. To overcome this, we introduce the expression graph network framework (EGNF). This cutting-edge graph-based approach integrates graph neural networks with network-based feature engineering to enhance the predictive identification of biomarkers. EGNF constructs biologically informed networks by combining gene expression data and clinical attributes within a graph database, utilizing hierarchical clustering to generate dynamic, patient-specific representations of molecular interactions. Leveraging graph learning techniques, including graph convolutional networks and graph attention networks, our framework identifies statistically significant and biologically relevant gene modules for classification. Validated across three independent datasets consisting of contrasting tumor types and clinical scenarios, EGNF consistently outperforms traditional machine learning models, achieving superior classification accuracy and interpretability. Notably, it delivers perfect separation between normal and tumor samples while excelling in nuanced tasks such as classifying disease progression and predicting treatment outcomes. This scalable, interpretable, and robust framework provides a powerful tool for biomarker discovery, with wide-ranging applications in precision medicine and the elucidation of disease mechanisms across diverse clinical contexts.

## Introduction

Classification problems constitute the cornerstone of machine learning, with the paramount objective of categorizing observations into discrete classes based on their attributes. This process is especially critical in healthcare, where precise classification has a direct impact on diagnosis, treatment strategies, and patient outcomes. Machine learning has revolutionized traditional analytical methods, offering innovative approaches to process and interpret complex datasets. The application of these techniques to biomarker discovery has emerged as a particularly promising domain, providing researchers with powerful tools to identify molecular indicators that can predict disease states, progression, and treatment response.

Cancer represents a complex disease characterized by the accumulation of genetic and epigenetic alterations that drive dysregulated gene expression, metabolic reprogramming, and aberrant cellular signaling pathways [1]. The molecular heterogeneity of cancer presents significant challenges for accurate classification and biomarker discovery, as tumors exhibit distinct gene expression profiles that vary both between and within cancer types. This complexity arises from the interconnected nature of biological pathways, where alterations in one molecular component can cascade through multiple regulatory networks, affecting cellular processes ranging from DNA repair mechanisms to immune response modulation. Traditional analytical approaches that treat genes as independent entities often fail to capture these intricate molecular interactions, highlighting the need for sophisticated methodologies that can model the network-based nature of cancer biology.

Isocytrate Dehydrogenase-wildtype (IDH-wt) glioblastoma exemplifies these challenges as the most aggressive and heterogeneous primary brain tumor in adults. Recent studies have revealed that IDH-wt glioblastomas exhibit profound molecular diversity with distinct gene expression subtypes that correlate with different clinical outcomes and treatment responses [2, 3]. The tumor microenvironment further contributes to this complexity, with significant intratumoral heterogeneity observed at the single-cell level, where different cellular populations within the same tumor display varied transcriptional programs [4]. This molecular complexity makes accurate subtype classification crucial for clinical decision-making, yet conventional machine learning approaches often struggle to identify robust biomarkers that can reliably distinguish between these molecular subtypes due to their inability to account for the interconnected nature of dysregulated pathways in glioblastoma pathogenesis.

Graph-based learning approaches have garnered significant attention in biomedical research due to their distinctive ability to model intricate relationships between biological entities. Unlike traditional machine learning methods that treat samples as independent observations, graph-based approaches leverage the inherent interconnectedness of biological data, capturing relationships that might otherwise remain obscured. This capability is particularly valuable in biomarker discovery, where understanding the interactions between molecules can provide more profound insights into disease mechanisms than analyzing individual features in isolation.

Cluster-based feature engineering is a powerful technique for enhancing machine learning models by grouping similar data points based on shared attributes, thereby enabling the extraction of meaningful patterns. One widely used method is the k-means clustering algorithm, which clusters data points based on proximity in feature space [5]. K-means clustering has been employed in various feature engineering applications to enhance model performance by identifying relationships between neighboring data points. By analyzing interrelationships within these clusters, models can make more informed predictions, as interactions between neighboring data points often reveal underlying data structures [6]. Unlike dimensionality reduction techniques, cluster-based methods like k-means clustering focus on enhancing model performance by capitalizing on these interrelationships rather than reducing feature count. Hierarchical clustering extends this concept by creating nested clusters at different levels of similarity, which aligns conceptually with the multi-level nature of biological interactions and provides a natural framework for discovering biomarker relationships across different scales of biological organization.

Among various classification techniques, traditional methods like logistic regression remain notable for their simplicity and interpretability in biomarker studies. Despite their widespread use, these methods often fail to capture the complex, non-linear relationships present in biological systems, thereby constraining their utility for comprehensive biomarker discovery. While support vector machines (SVMs), random forest models, and elastic net regression offer more robust approaches to biomarker discovery, especially in high-dimensional settings, these methods primarily operate on tabular data and do not inherently account for the network structure of biological systems. Random forest models, though capable of capturing non-linear interactions through their ensemble of decision trees, still treat features independently and cannot directly incorporate the relational information encoded in biological networks.

Graph neural networks (GNNs) have emerged as a powerful class of models designed to advance biomarker discovery by leveraging graph-structured data prevalent in biological applications. GNNs capture complex relationships between biological entities represented as nodes, along with their interactions defined by edges. Among the most prominent GNN architectures are graph convolutional networks (GCNs) and graph attention networks (GATs). GCNs extend convolutional neural networks to graph data by leveraging the adjacency structure, enabling efficient information propagation among connected features [7]. GATs enhance this capability by introducing attention mechanisms that allow the model to dynamically weigh the importance of different node neighbors [8]. More recent architectures further improve performance through positional and structural encodings that better capture the graph topology [9].

The integration of multi-omics data through graph-based approaches has significantly enhanced the identification of clinically relevant biomarkers. Previous studies have demonstrated superior performance in patient classification and biomarker identification compared to conventional methods. For instance, Wang et al. [10] introduced MOGONET, a framework integrating multi-omics data using GCNs. Similarly, Ramirez et al. [11] applied GCNs to cancer classification, showcasing the potential of graph-based approaches in distinguishing between cancer types based on gene expression data. These studies highlight the power of graph-based methods in capturing the complex interplay between different biological layers.

Existing graph-based approaches for biomarker discovery typically rely on established biological networks, such as protein-protein interaction networks or co-expression networks. The weighted gene co-expression network analysis (WGCNA) developed by Langfelder and Horvath [12] has been widely used to identify modules of co-expressed genes that may serve as potential biomarkers. Building upon this foundation, recent approaches have integrated deep learning architectures with network-based feature extraction to enhance biomarker identification. For instance, Yu et al. [13] proposed iHofman, which combines hierarchical autoencoders with weighted attention mechanisms for circRNA–miRNA interaction prediction, demonstrating how attention-based fusion of sequence and structural features can improve predictive performance. Similarly, advanced GNN approaches have incorporated multi-level attention mechanisms and feature fusion strategies to capture complex biological relationships [14, 15]. These multi-level attention graph neural networks based on co-expression gene modules have shown promise for disease diagnosis and prognosis, demonstrating improved predictive performance and interpretability over traditional network-based methods.

Despite these advances, a critical limitation of existing approaches is that they are not specifically tailored for tissue sample classification in biomarker discovery. Most methods rely on predefined biological networks, which may not accurately reflect the specific relationships relevant to the disease or condition under investigation. Additionally, these approaches often struggle to handle datasets with varying sample sizes, limiting their applicability across different clinical contexts.

This research advances machine learning in biomedical applications through two primary contributions. First, we develop the expression graph network framework (EGNF), a novel methodology integrating network generation with GCNs and GATs for gene expression-based classification. EGNF leverages deep learning to enhance the extraction of complex patterns and relationships from gene expression data, significantly improving classification accuracy. The generated networks capture intricate relationships between samples and features, adaptively configuring to different sample sizes while preserving biological relevance. Our approach uniquely employs hierarchical clustering to identify meaningful biological relationships, providing a natural bridge between conventional cluster-based feature engineering and advanced graph-based learning methods.

Second, we develop a biologically meaningful network-based feature selection method specifically designed for gene expression data. By combining network analysis with conventional statistical techniques, our method identifies gene modules that are both statistically significant and biologically relevant, offering more profound insights into disease progression mechanisms. This approach reduces data complexity while maintaining predictive power, ultimately improving the interpretability of machine learning models.

Together, these contributions offer several advantages over existing methods: (i) they enable more accurate patient/sample stratification by leveraging complex patterns encoded in generated graphs; (ii) they provide insights into biological mechanisms underlying disease states by highlighting important connections between biomarkers; (iii) they facilitate the integration of multi-modal data, capturing relationships spanning different biological domains; and (iv) they demonstrate robust performance across different datasets and disease types, suggesting broad applicability in precision medicine.

## Materials and methods

### Overview of EGNF

**Figure 1:**
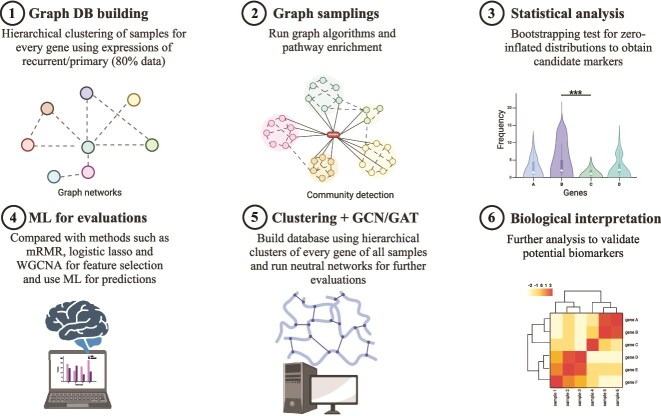
Framework for network-based feature selection.

Our methodological framework consists of several sequential analytical stages (Fig. 1). Initially, we performed differential expression analysis on 80% of the data using DESeq2 to identify differentially expressed genes [16]. Using this training data, we constructed a graph network by selecting extreme sample clusters with high or low median expression for each group (unpaired method), or group ratio values (paired method) from one-dimensional hierarchical clustering as nodes and establishing connections between sample clusters of different genes through shared samples. We then conducted graph-based feature selection considering three criteria: node degrees, gene frequency within communities, and inclusion in known biological pathways. The selected features were then used to generate sample clusters via one-dimensional hierarchical clustering, which served as nodes for building the prediction network. In the final stage, we utilized GNNs for sample-specific graph-based predictions, where each sample was represented by a corresponding subgraph structure.

This study utilized open-source libraries for biomarker discovery, including PyTorch Geometric for GNN model development and network analysis tools, such as Neo4j and their Graph Data Science (GDS) library. All algorithm development and validation phases were executed within this computational framework to ensure reproducibility and scalability.

### Datasets

This study used three paired gene expression datasets to assess model performance across clinical contexts. The glioma dataset comprised 295 primary and 275 corresponding recurrent tumors from IDH-wt patients, sourced from the Glioma Longitudinal Analysis Consortium and the MD Anderson Cancer Center’s Glioblastoma Moon Shot project, enabling analysis of molecular changes during disease progression. The breast cancer dataset included 111 normal tissue specimens paired with 113 matched tumor samples from The Cancer Genome Atlas Program, allowing for the examination of differences between healthy and malignant tissues. The third dataset contained 69 matched pre- and post-treatment samples from HER2-negative breast cancer patients who underwent neoadjuvant chemotherapy with Bevacizumab (GSE87455), obtained from the GEO repository, facilitating analysis of treatment-induced molecular alterations. All datasets exclusively included patients with complete paired gene expression profiles; unpaired samples were excluded from the analysis.

### Network generation

**Figure 2:**
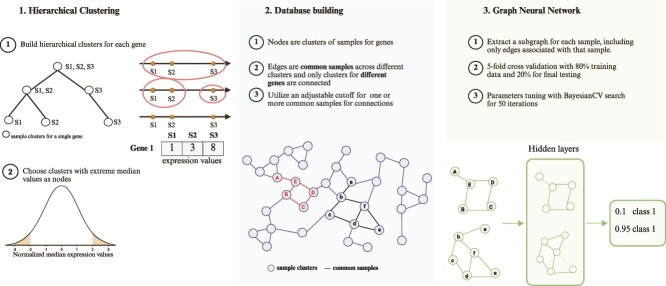
Network generation process.

After normalization using dataset-specific approaches, we performed gene-wise hierarchical clustering using a bottom-up agglomerative approach with Euclidean distance and median linkage. For paired datasets, we applied $\log _{2}(x+1)$ normalization followed by rescaling values to a range of 1 to 2 for each sample class, then used the class2/class1 ratios to perform one-dimensional hierarchical clustering. For unpaired datasets, we employed $\log _{2}(x+1)$ normalization followed by z-normalization for each sample class and conducted hierarchical clustering separately. This median merge provides robust clustering by reducing sensitivity to outliers and extreme values compared to single or complete linkage, enabling the identification of distinct sample clusters (Fig. 2). From the resulting clusters for each gene, we selected the top 10% most extreme clusters based on absolute z-normalized median expression values to serve as nodes in our gene-gene interaction network, constructed using the Neo4j graph database. Each node represents a gene with associated properties including median expression value, gene identifier, sample count, and levels within trees. Edges connect genes sharing samples between clusters, with edge formation controlled by a minimum common sample threshold (default = 1), and each edge annotated with the number of shared samples and tree levels of connected nodes. Sample-specific subgraphs were extracted by identifying all relevant nodes and edges for each sample, providing structured input features for downstream GNN classification tasks. Prior to GNN model training, all node features and edge features were normalized to ensure consistent scaling and optimal convergence during the learning process.

### Graph-based feature selection for identifying biomarkers

We developed network-based feature selection methods for identifying significant biomarkers in paired and unpaired datasets. Both approaches begin with dimension reduction using DESeq2, followed by dataset-specific normalization. We then applied the above hierarchical clustering and network construction method above to build the gene-gene interaction graph.

To reduce the impact of indirect, complex relationships among the same gene, we randomly selected a single sample cluster for each gene per graph sampling iteration and repeated this process 10 000 times. Degree centrality algorithms were applied to enumerate degrees for each gene. Modularity optimization algorithms were utilized to delineate gene communities. For each community, we quantified gene frequency and scrutinized whether genes within communities are incorporated in enriched pathways. Bootstrapping analyses further refined marker selection by comparing each marker’s mean expression against the mean expression of all alternative markers, addressing the zero-inflated distribution of degree, frequency, and pathway scores.

To evaluate the impact of pathway enrichment on predictive performance, we constructed reference marker catalog devoid of pathway enrichment. A comprehensive scoring system was developed to prioritize markers by ranking adjusted p-values according to various filtration criteria and employing a summing rank for definitive selection.

### Other feature selection methods

Weighted Gene Correltaion Network Analysis (WGCNA), Analysis of Variance (ANOVA), and Minimum Redundency Maximum Relevance (mRMR) are three complementary feature selection methods used to identify informative genes from high-dimensional gene expression data. WGCNA constructs gene co-expression networks by clustering genes based on their correlation patterns, identifying modules of highly correlated genes, and associating them with phenotypic traits to uncover biologically relevant relationships. ANOVA, a statistical approach, determines whether gene expression levels significantly differ across multiple groups by comparing within-group and between-group variability. Lastly, mRMR selects features that are both highly relevant to the target variable and minimally redundant with each other, ensuring an informative yet non-redundant subset of genes for downstream analysis. These methods collectively enhance the robustness of feature selection by integrating network-based, statistical, and information-theoretic approaches.

### Graph convolutional network model

GCNs extend the concept of convolutional operations to graph-structured data. A GCN model operates by aggregating feature information from neighboring nodes in a graph to learn node representations. Given a graph $ G = (V, E) $ where $ V $ represents the set of nodes and $ E $ is the set of edges, let $ A $ be the adjacency matrix. The propagation rule of a GCN layer can be expressed as:


\begin{align*} & H^{(l+1)} = \sigma(\tilde{D}^{-1/2} \tilde{A} \tilde{D}^{-1/2} H^{(l)} W^{(l)}) \end{align*}

where $\tilde{A} = A + I_{N}$ is the adjacency matrix with added self-connections, $\tilde{D}$ is the diagonal degree matrix, $H^{(l)}$ is the node feature matrix in layer $l$, $W^{(l)}$ is the learnable weight matrix in layer $l$, and $\sigma (\cdot )$ is a non-linear activation function such as ReLU.

### Graph attention network model

GATs introduce attention mechanisms into GNNs, allowing the model to assign different importance weights to different neighbors. The attention coefficient between node $i$ and neighbor $j$ is computed as:


\begin{align*} & e_{ij} = \text{LeakyReLU}(a^{T} [W^{(l)} H_{i}^{(l)} \, || \, W^{(l)} H_{j}^{(l)}]) \end{align*}

where $H_{i}^{(l)}$ and $H_{j}^{(l)}$ are the input feature vectors of nodes $i$ and $j$ for layer $l$, $W^{(l)}$ is a learnable weight matrix, $a$ is a learnable attention vector, || denotes concatenation, and LeakyReLU is an activation function. The attention coefficients are normalized using the softmax function across all neighbors $j$ of node $i$:


\begin{align*} & \alpha_{ij} = \frac{\exp(e_{ij})}{\sum_{k \in \mathcal{N}(i)} \exp(e_{ik})} \end{align*}

The node feature update for node $i$ is then computed as a weighted sum of its neighbors’ features:


\begin{align*} & H^{(l+1)} = \sigma\left(\sum_{j \in \mathcal{N}(i)} \alpha_{ij} W^{(l)} H_{j}^{(l)}\right) \end{align*}

Where $\sigma $ is a non-linear activation function, and $\alpha _{ij}$ are the attention coefficients that weight the contribution of each neighbor’s features. GCNs aggregate neighborhood information uniformly using normalized adjacency matrices, while GATs utilize an attention mechanism to focus on the most relevant neighbors, dynamically weighting each neighbor’s contribution to a node’s feature representation.

### Graph attention network v2 model

Graph attention network v2 (GATv2) improves upon the original GAT by introducing dynamic attention mechanisms that better capture node relationships [9]. Unlike GAT, where the attention coefficients are computed in a static manner, GATv2 allows for a more expressive and adaptive attention function by making the attention mechanism order-invariant. This enables the model to assign attention to weights dynamically based on node features rather than relying on predefined structures.

The attention mechanism in GATv2 is defined similarly to GAT but introduces a critical modification. For a node, the attention coefficient between node and one of its neighbors is computed as:


\begin{align*} & e_{ij} = a^{T} \text{LeakyReLU}(W^{(l)} (H_{i}^{(l)} || H_{j}^{(l)})) \end{align*}

Beyond this modification, the normalization of attention coefficients and the node feature update follow the same formulation as in GAT. By leveraging this improved attention mechanism, GATv2 provides better adaptability and expressiveness in learning from graph-structured data.

### Traditional machine learning models

Traditional machine learning approaches have shown considerable success in various classification tasks. Logistic regression, a fundamental statistical model, provides interpretable results by modeling probability through a logistic function [17]. The elastic net extends conventional regression by incorporating both L1 and L2 regularization, effectively handling multicollinearity and performing feature selection simultaneously [18]. Random Forest, an ensemble learning method, combines multiple decision trees to reduce overfitting and improve generalization by leveraging bagging and random feature selection [19]. SVM excels in finding optimal hyperplanes to separate classes in high-dimensional spaces through kernel transformations [20]. Multilayer Perceptron (MLP), a type of artificial neural network, can capture complex non-linear relationships by utilizing multiple layers of interconnected neurons with activation functions [21]. These models have been widely applied across various domains, demonstrating their versatility and effectiveness in handling different types of data and classification problems.

### Parameter tuning process

Model hyperparameters were optimized using five-fold cross-validation on the training dataset. For conventional machine learning models (logistic regression, elastic net, random forest, SVM, and MLP), we employed random search to efficiently explore parameter spaces [22], optimizing regularization parameters for the elastic net, tree parameters for random forest, kernel parameters for SVM, and network architecture for MLP. For GNNs, we utilized Bayesian optimization to navigate their more complex parameter spaces, including network depth, learning rates, and attention mechanisms [23]. Conventional machine learning analyses were implemented using the caret package [24] in R with parallel processing, while GNN computations were performed on an NVIDIA GPU with 45 GB VRAM in an HPC environment. All models were evaluated through ten iterations of training and testing, with the final performance assessed using average accuracy and AUC scores.

## Results

### GNN-based classification improves performance

**Figure 3:**
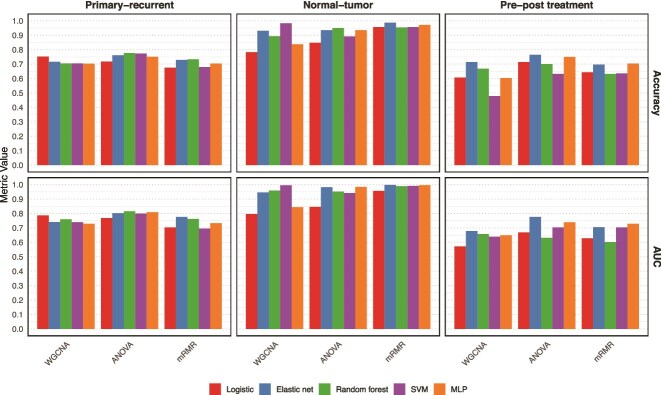
Performance of machine learning models for different features.

The machine learning models, trained on gene expression profiles selected through WGCNA, mRMR, and ANOVA, displayed varied performance across different classification tasks (Fig. 3). They showed strong results in binary classification of normal versus tumor samples in breast cancer, achieving high accuracy and AUC. However, their performance decreased in more complex tasks like distinguishing primary from recurrent tumors and pre- from post-treatment samples.

We implemented our methodology incorporating network generation and GNNs and compared its performance against traditional machine learning models (Fig. 3) using accuracy and AUC as baselines across 10 iterations. Table 1 presents the relative improvements achieved by our GNN-based classification for each feature set. Our method enhanced accuracy by 13.8%, −2.4%, and 2.0% across the three datasets. Similarly, it improved AUC by 17.1%, −0.7%, and 1.3%, respectively. The variability in magnitude improvement is likely due to differences in sample sizes and the complexity of the classification task. GNNs faced challenges in achieving superior performance in data-limited contexts.

**Table 1:** Performance comparison across datasets and feature selection methods

**Dataset**	**Features**	**Accuracy (SD)**				**AUC (SD)**			
		**GCN-gene**	**GCN**	**GAT**	**GATv2**	**GCN-gene**	**GCN**	**GAT**	**GATv2**
Primary-recurrent	WGCNA	0.717 (0.017)	0.748 (0.058)	0.698 (0.063)	0.740 (0.008)	0.780 (0.013)	0.782 (0.085)	0.731 (0.088)	0.771 (0.011)
	ANOVA	0.838 (0.006)	0.847 (0.034)	0.855 (0.017)	0.850 (0.020)	0.901 (0.005)	0.897 (0.032)	0.901 (0.019)	0.900 (0.024)
	mRMR	**0.884 (0.010)**	0.820 (0.056)	0.866 (0.021)	0.862 (0.020)	**0.954 (0.004)**	0.892 (0.055)	0.932 (0.018)	0.924 (0.009)
Normal-tumor	WGCNA	0.813 (0.027)	**0.963 (0.023)**	0.917 (0.030)	0.922 (0.026)	0.806 (0.014)	**0.991 (0.008)**	0.961 (0.020)	0.967 (0.017)
	ANOVA	0.583 (0.073)	0.943 (0.046)	0.865 (0.132)	0.913 (0.023)	0.399 (0.191)	0.978 (0.032)	0.907 (0.144)	0.950 (0.015)
	mRMR	0.746 (0.143)	0.935 (0.010)	0.928 (0.031)	0.952 (0.009)	0.712 (0.122)	0.976 (0.004)	0.968 (0.009)	0.974 (0.015)
Pre-post treatment	WGCNA	-	0.700 (0.018)	0.696 (0.019)	0.696 (0.019)	-	0.649 (0.007)	0.647 (0.005)	0.645 (0.006)
	ANOVA	-	0.721 (0.058)	0.679 (0.041)	0.700 (0.063)	-	0.733 (0.076)	0.646 (0.052)	0.680 (0.081)
	mRMR	-	**0.779 (0.037)**	0.675 (0.070)	0.689 (0.034)	-	**0.786 (0.036)**	0.660 (0.069)	0.671 (0.043)

**Bold values** indicate the best performance for each dataset.

### Computational cost of GNNs

Table 2 documents the computational time for each model across different datasets. The GNN-based approaches required significantly more computational resources compared to traditional machine learning models. However, enhanced classification performance can justify this increased computational cost. Notably, our GNN implementations were run sequentially due to resource limitations; parallel processing could potentially lead to substantial improvements in computational efficiency.

**Table 2:** Time cost for different models across tasks

**Model**	**Primary-recurrent**	**Normal-tumor**	**Pre-post treatment**
Logistic	0.5 s	0.5 s	0.4 s
Elastic net	1.7 s	3.1 s	1.2 s
Random forest	15.6 s	2.6 s	1.6 s
SVM	6.8 s	5.0 s	1.9 s
MLP	13.7 s	7.4 s	3.1 s
GCN-gene	1$\sim $2 days	2$\sim $4 h	-
GCN-all-feature	1$\sim $2 days	2$\sim $4 h	2$\sim $3 h
GAT-all-feature	2$\sim $3 days	3$\sim $6 h	3$\sim $6 h

### Graph-based features enhance performance

**Figure 4:**
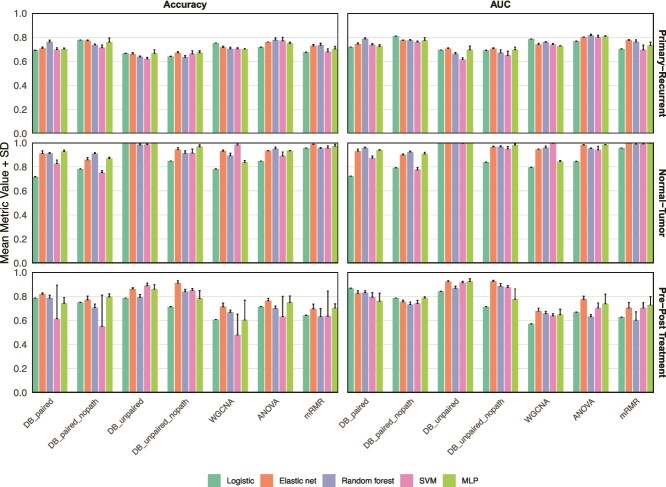
Performance of different features including graph-based features.

We utilized graph-based feature profiles to construct graph networks. The GNN predictions leveraging these networks demonstrated superior performance compared to all other feature types (Fig. 4). In traditional machine learning models, graph-based features achieved perfect separation in the normal-tumor classification task and exceeded 90% accuracy and AUC in pre- and post-treatment sample classification. These features also delivered competitive performance for primary-recurrent classification.

The GNN models achieved an accuracy of 0.948 and an AUC of 0.977 in the primary-recurrent dataset, perfect separation in the normal-tumor dataset, and an accuracy of 0.896 with an AUC of 0.926 in the pre-post treatment dataset (Table 3). With our feature selection methodology, accuracy improved by 7.2%, 1.3%, and 16.9% in these respective tasks, while AUC increased by 2.4%, 0.2%, and 17.8%. The exceptional performance of DB_unpaired and DB_unpaired_nopath features in GNN models highlights the effectiveness of our approach in capturing complex biological relationships.

**Table 3:** Extended performance comparison with paired/unpaired datasets and feature selection methods

**Dataset**	**Features**	**Accuracy (SD)**				**AUC (SD)**			
		**GCN-gene**	**GCN**	**GAT**	**GATv2**	**GCN-gene**	**GCN**	**GAT**	**GATv2**
Primary-recurrent	DB_Paired	0.816 (0.023)	0.775 (0.047)	0.733 (0.033)	0.779 (0.014)	0.882 (0.014)	0.833 (0.041)	0.785 (0.042)	0.843 (0.019)
	DB_paired_nopath	0.821 (0.015)	0.812 (0.037)	0.733 (0.025)	0.784 (0.052)	0.896 (0.014)	0.872 (0.035)	0.773 (0.036)	0.837 (0.063)
	DB_unpaired	0.938 (0.048)	0.942 (0.014)	0.915 (0.027)	0.908 (0.016)	0.977 (0.031)	**0.977 (0.015)**	0.963 (0.015)	0.958 (0.014)
	DB_unpaired_nopath	**0.948 (0.016)**	0.937 (0.023)	0.910 (0.023)	0.928 (0.019)	0.971 (0.014)	0.974 (0.024)	0.961 (0.013)	0.970 (0.012)
	WGCNA	0.717 (0.017)	0.748 (0.058)	0.698 (0.063)	0.740 (0.008)	0.780 (0.013)	0.782 (0.085)	0.731 (0.088)	0.771 (0.011)
	ANOVA	0.838 (0.006)	0.847 (0.034)	0.855 (0.017)	0.850 (0.020)	0.901 (0.005)	0.897 (0.032)	0.901 (0.019)	0.900 (0.024)
	mRMR	0.884 (0.010)	0.820 (0.056)	0.866 (0.021)	0.862 (0.020)	0.954 (0.004)	0.892 (0.055)	0.932 (0.018)	0.924 (0.009)
Normal-tumor	DB_paired	0.617 (0.104)	0.911 (0.036)	0.889 (0.047)	0.899 (0.041)	0.492 (0.264)	0.944 (0.033)	0.887 (0.031)	0.919 (0.040)
	DB_paired_nopath	0.580 (0.060)	0.902 (0.021)	0.828 (0.033)	0.885 (0.069)	0.457 (0.168)	0.940 (0.007)	0.834 (0.034)	0.903 (0.077)
	DB_unpaired	0.815 (0.033)	0.980 (0.007)	**1.000 (0.000)**	0.985 (0.034)	0.832 (0.048)	0.995 (0.004)	**1.000 (0.000)**	0.993 (0.018)
	DB_unpaired_nopath	0.717 (0.093)	0.948 (0.011)	0.835 (0.122)	0.889 (0.019)	0.693 (0.121)	0.960 (0.004)	0.854 (0.132)	0.917 (0.034)
	WGCNA	0.813 (0.027)	0.963 (0.023)	0.917 (0.030)	0.922 (0.026)	0.806 (0.014)	0.991 (0.008)	0.961 (0.020)	0.967 (0.017)
	ANOVA	0.583 (0.073)	0.943 (0.046)	0.865 (0.132)	0.913 (0.023)	0.399 (0.191)	0.978 (0.032)	0.907 (0.144)	0.950 (0.015)
	mRMR	0.746 (0.143)	0.935 (0.010)	0.928 (0.031)	0.952 (0.009)	0.712 (0.122)	0.976 (0.004)	0.968 (0.009)	0.974 (0.015)
Pre-post	DB_paired	-	0.793 (0.028)	0.721 (0.041)	0.711 (0.064)	-	0.833 (0.030)	0.740 (0.071)	0.691 (0.064)
	DB_paired_nopath	-	0.793 (0.073)	0.718 (0.052)	0.696 (0.030)	-	0.789 (0.080)	0.698 (0.057)	0.669 (0.050)
	DB_unpaired	-	0.846 (0.041)	0.850 (0.015)	0.843 (0.066)	-	0.892 (0.034)	0.886 (0.019)	0.858 (0.092)
	DB_unpaired_nopath	-	0.796 (0.024)	0.871 (0.063)	**0.896 (0.057)**	-	0.839 (0.032)	0.915 (0.053)	**0.926 (0.057)**
	WGCNA	-	0.700 (0.018)	0.696 (0.019)	0.696 (0.019)	-	0.649 (0.007)	0.647 (0.005)	0.645 (0.006)
	ANOVA	-	0.721 (0.058)	0.679 (0.041)	0.700 (0.063)	-	0.733 (0.076)	0.646 (0.052)	0.680 (0.081)
	mRMR	-	0.779 (0.037)	0.675 (0.070)	0.689 (0.034)	-	0.786 (0.036)	0.660 (0.069)	0.671 (0.043)

**Bold values** indicate the best performance for each dataset.

### Biomarkers selected from unpaired approach

Our approach successfully identified key predictive markers from unpaired feature selection, many of which have literature support for their functional relevance (Supplementary Table S1). In IDH-wt glioma, PGR, COL17A1, and RGS14 showed strong predictive value for recurrence, consistent with their known roles in tumor aggressiveness and therapy resistance [25–27]. For breast cancer normal-tumor differentiation, CXCL14, MSLN, LCN2, and MED1 emerged as significant predictors, supported by evidence of their differential expression and involvement in tumor progression [28–31]. In HER2-negative breast cancer treated with Bevacizumab, MMP2, COL5A2, COL6A1, DLK1, and MDK were identified as predictive of treatment response, aligning with their documented roles in angiogenesis and stromal interactions [32–36]. These findings validate our biomarker discovery method and underscore the biological relevance of the identified markers, enhancing their potential translational value in precision oncology.

## Discussion

The EGNF leverages the power of GNNs to advance biomarker identification through gene expression data, offering a robust approach to modeling biological relationships. To the best of our knowledge, this represents the first time that common samples have been used to connect nodes in a biological network instead of relying on traditional logical relationships such as protein-protein interactions or pathway memberships. This novel approach enables the discovery of functional relationships that may not be captured by existing biological databases, potentially revealing new therapeutic targets and diagnostic markers (Table 4). The predictive markers identified by EGNF across the three datasets—IDH-wt glioma, normal-tumor breast cancer, and HER2-negative breast cancer treated with Bevacizumab—exemplify the model’s ability to uncover biologically significant genes within complex expression networks.

**Table 4:** Featured biomarkers identified by EGNF across cancer datasets with supporting clinical evidence

**Cancer type**	**Biomarker**	**Biological function**	**Clinical evidence**
IDH-wt glioma	PGR	Progesterone signaling/migration	[25, 37]
	COL17A1	ECM remodeling/invasion	[26]
	RGS14	Immune regulation/prognosis	[27]
Breast cancer (normal-tumor)	CXCL14	Anti-cancer/immune modulation	[29, 40–42]
	MSLN	Immunotherapy target	[30]
	LCN2	Metastasis promotion	[28]
	MED1	Estrogen resistance	[31]
HER2-negative breast cancer (Bevacizumab)	MMP2	Matrix degradation/invasion	[32]
	COL5A2	Tumor microenvironment	[34]
	COL6A1	Prognosis/microenvironment	[34]
	DLK1	NOTCH signaling modulation	[33]
	MDK	Angiogenesis/therapy resistance	[35, 36]

In IDH-wt glioma, our approach identified PGR, COL17A1, and RGS14 as key predictive markers for recurrence, consistent with their known roles in tumor aggressiveness and therapy resistance. These markers reflect pathways associated with recurrence, including progesterone signaling, extracellular matrix remodeling, and G-protein regulation, respectively, which are critical in the biology of aggressive gliomas. Recent clinical studies have further validated these findings: PGRMC1 has been demonstrated as a tumor-promoting factor in glioblastoma, where it modulates tumor progression, immune microenvironment, and therapy response [25, 37]. COL17A1’s role in ECM remodeling and invasion has been supported by integrated analyses identifying it as a potential biomarker of glioblastoma multiforme [26], while RGS14’s involvement in immune regulation and prognosis has been confirmed through machine learning studies unveiling immune-related signatures in multicenter glioma research [27]. Recurrence represents a major clinical challenge in glioma management, with IDH-wt tumors showing particularly aggressive behavior and high recurrence rates, often leading to treatment failure and poor patient outcomes [38, 39]. The identification of molecular signatures associated with recurrence could enable earlier intervention and personalized treatment strategies, potentially improving the dismal prognosis currently associated with these tumors.

For breast cancer normal-tumor differentiation, CXCL14, MSLN, LCN2, and MED1 emerged as significant predictors, supported by evidence of their differential expression and involvement in tumor progression. These markers highlight immune modulation, cell surface alterations, inflammation, and estrogen receptor coactivation, processes central to tumorigenesis. The clinical relevance of these biomarkers has been extensively validated: CXCL14 expression in tumor stroma has been confirmed as an independent survival marker, with fibroblast-derived CXCL14 promoting epithelial-to-mesenchymal transition and metastasis through ACKR2-dependent mechanisms [29, 40, 41]. Importantly, low CXCL14 expression levels have been specifically associated with poor survival rates in triple-negative breast cancer patients [42]. MSLN has been validated as a novel immunotherapy target for triple negative breast cancer [30], while LCN2’s role in promoting breast cancer progression and metastasis has been well-documented [28]. MED1’s involvement in estrogen resistance mechanisms has been demonstrated, where silencing MED1 sensitizes breast cancer cells to pure anti-estrogen fulvestrant both in vitro and in vivo [31].

In the HER2-negative breast cancer cohort treated with Bevacizumab, MMP2, COL5A2, COL6A1, DLK1, and MDK were identified as predictive of treatment response, aligning with their documented roles in angiogenesis and stromal interactions. These markers demonstrate the regulation of angiogenesis, stromal architecture, and growth factor signaling pathways. Treatment resistance in breast cancer, particularly to targeted therapies like Bevacizumab, remains a significant obstacle in clinical management, with most patients eventually developing resistance mechanisms that lead to disease progression [43]. The clinical validation of these markers is substantial: MMP2 values in tumor tissue have been characterized in basal-like breast cancer patients, demonstrating their role in matrix degradation and invasion [32]. COL5A2 and COL6A1 have been identified through pan-cancer analyses as significant factors in prognosis and tumor microenvironment modulation [34]. DLK1’s different expression levels have been shown to inversely modulate the oncogenic potential of breast cancer cells through inhibition of NOTCH1 signaling [33]. MDK’s role in angiogenesis and therapy resistance has been confirmed through angiogenesis-related analyses associated with prognosis and tumor immune microenvironment, as well as single-cell RNA sequencing studies identifying molecular biomarkers predicting progression to CDK4/6 inhibition [35, 36]. MMP2 and collagen genes influence extracellular matrix dynamics affecting drug delivery, while MDK provides an alternative angiogenic signaling pathway that may contribute to treatment resistance. These markers represent interconnected biological processes that determine Bevacizumab response, offering insights for patient stratification in anti-angiogenic therapy and enabling clinicians to identify patients who might benefit from alternative or combination approaches before resistance develops.

It is important to note that markers not listed in Table 4 still warrant further investigation, even in the absence of direct supporting clinical studies. The novel network construction approach employed by EGNF may reveal previously uncharacterized gene interactions and functional relationships that have not yet been explored in clinical contexts. These markers could represent emerging therapeutic targets or novel biomarkers that require validation through future experimental and clinical studies.

### Limitations

While EGNF demonstrates significant promise, several limitations must be acknowledged. Interpretability remains a critical focus in GNN research, aligning with EGNF’s emphasis on biologically meaningful feature selection. Advances have introduced GNN models with explainability layers that highlight key gene subnetworks driving predictions in cancer prognosis [44, 45]. This approach not only improves classification but also provides clinicians with actionable insights into disease mechanisms. Integrating such interpretability mechanisms into EGNF could enhance its ability to identify significant gene modules, thereby increasing its value for translational research, where biological relevance is paramount.

Scalability is another consideration, as GNNs often require substantial computational resources. Research has demonstrated that optimization techniques, such as pruning redundant graph connections, can reduce computational overhead without sacrificing accuracy in gene expression tasks [46]. GNN model performance could be further enhanced through two key optimizations: (i) implementation of a more comprehensive hyperparameter search strategy, and (ii) adoption of more stringent thresholds for shared sample selection. These potential enhancements suggest that the current performance metrics of GNNs represent a conservative estimate of the methodology’s capabilities. For EGNF, adopting such optimization strategies could make it more feasible for large-scale genomic studies, broadening its practical utility.

### Ethical considerations

The deployment of biomarker discovery pipelines like EGNF in translational medicine raises important ethical implications that must be carefully considered. First, the potential for algorithmic bias in biomarker selection could lead to disparities in healthcare outcomes if training datasets are not representative of diverse patient populations. Ensuring equitable representation across different ethnic groups, socioeconomic backgrounds, and geographic regions is crucial for developing universally applicable biomarkers. Second, the clinical implementation of Artificial Intelligence (AI) derived biomarkers requires robust validation frameworks to prevent premature adoption of markers that may lack sufficient clinical evidence. The integration of such biomarkers into clinical decision-making must be accompanied by appropriate regulatory oversight and transparency in algorithmic processes to maintain patient trust and safety. Additionally, questions of data ownership, patient consent for AI-driven analysis, and the potential commercialization of biomarker discoveries necessitate clear ethical guidelines and governance frameworks. Finally, the accessibility and affordability of biomarker-based diagnostics and treatments must be considered to prevent exacerbation of existing healthcare inequalities.

### Future directions

Recent developments in the field offer valuable insights into how GNN-based methods, such as EGNF, can be contextualized and potentially enhanced. One notable trend is the application of GNNs to multi-modal biological data for improved disease classification and biomarker discovery. Researchers have explored hybrid GNN models that integrate gene expression profiles with protein-protein interaction networks to identify cancer-specific biomarkers, achieving high predictive accuracy by capturing cross-modal dependencies [47]. This suggests that EGNF could benefit from incorporating additional data types, such as epigenetic or proteomic information, to enrich its network representations. The ability to model such complex interactions is a key advantage of GNNs, enabling the detection of subtle patterns that traditional methods might overlook.

Another area of progress is the application of GNNs to rare disease research, where data scarcity presents a significant challenge. Studies have shown that GNN-based frameworks, combined with transfer learning, can effectively identify biomarkers using limited gene expression datasets [48, 49]. This is particularly relevant for EGNF, as its performance in scenarios with small sample sizes, such as distinguishing nuanced disease states, could be bolstered by adopting similar techniques. By pre-training on larger, related datasets and fine-tuning for specific tasks, EGNF may overcome limitations associated with data availability, thereby enhancing its applicability in precision medicine.

In our future work, we will sequence samples for single-cell spatial transcriptomics analysis to provide complementary validation of our findings. This approach will enable biomarker characterization at cellular resolution within the tissue microenvironment, potentially revealing cell-type-specific signatures and spatial expression patterns that could enhance the clinical utility of the identified markers. Furthermore, it will allow us to assess whether the sample-based network connections inferred by EGNF reflect genuine cellular interactions and spatial organization within the tumor. In addition, recent studies have introduced controlled noise injection into the data to better manage false discovery rates, thereby yielding more reliable biomarker candidates [50]. Adopting such techniques could further strengthen the validity of markers identified by EGNF.

## Conclusion

Importantly, EGNF demonstrates two significant advantages that extend beyond our specific test cases. First, it offers a powerful approach for biomarker identification, extracting genes with genuine biological relevance rather than statistical artifacts. This capability stems from the method’s integration of network topology with expression data, enabling it to capture complex gene interactions that traditional feature selection methods might miss. Second, EGNF provides superior predictive performance across various classification tasks, as evidenced by our comparative analyses. This dual strength, identifying meaningful biomarkers while enhancing prediction accuracy, makes EGNF particularly valuable in clinical applications where both mechanistic insights and reliable patient stratification are crucial. Moreover, these capabilities suggest that EGNF could be effectively applied to any sample classification task involving gene expression data, regardless of the disease context or research area.

Looking ahead, EGNF is well positioned to benefit from emerging trends in GNN research, including multi-modal integration, transfer learning for small datasets, enhanced interpretability, and computational optimization. Incorporating these advances could further strengthen its robustness, broaden its applicability across disease contexts, and increase its clinical utility. Ultimately, EGNF has the potential to bridge the gap between computational innovation and clinical impact, offering a powerful tool for addressing critical challenges such as glioma recurrence and breast cancer treatment resistance.

**Key Points**

- We introduce a novel graph-based biomarker discovery framework that integrates graph neural networks (GNNs) with network-driven feature engineering, enabling biomarker identification by modeling gene expression as graph-structured data rather than independent features.
- The framework demonstrates superior predictive performance compared to traditional machine learning approaches by leveraging GNNs’ ability to capture complex gene-gene interactions and regulatory relationships within biological networks.
- Network-based features provide direct biological interpretability, allowing researchers to trace how specific graph structures and connectivity patterns contribute to biomarker significance and revealing insights into underlying disease mechanisms.
- The pipeline offers broad cross-disease generalizability, demonstrating robust transferability across diverse datasets and biological contexts for discovering context-specific biomarkers.
- This approach represents a paradigm shift from conventional feature selection methods toward network topology analysis, opening new avenues for understanding complex disease mechanisms through graph-structured biological data.

## Supplementary Material

Supplementary_Table_1_bbaf559

## Data Availability

The gene expression datasets used in this study are publicly available from the GLASS Consortium (https://www.synapse.org/Synapse:syn17038081/wiki/585622), TCGA (https://www.cancer.gov/ccg/research/genome-sequencing/tcga), and the GEO under accession number GSE87455 (https://www.ncbi.nlm.nih.gov/geo/query/acc.cgi?acc=GSE87455).

## Code availability

The source code and tutorial of this work can be found on GitHub https://github.com/yliu38/EGNF.
